# Biological and Targeted Therapy in Non-Infectious Uveitis: Current Evidence and Future Perspectives

**DOI:** 10.3390/life16091522

**Published:** 2026-09-13

**Authors:** Vesela Todorova Mitkova-Hristova, Marin Anguelov Atanassov, Ivaylo Atanasov Popov

**Affiliations:** 1 Department of Ophthalmology, Faculty of Medicine, Medical University of Plovdiv, 4002 Plovdiv, Bulgaria; marin.atanassov@mu-plovdiv.bg; 2 Clinic of Ophthalmology, University General Hospital “St. George”, 4001 Plovdiv, Bulgaria; 3 Department of Propedeutics of Internal Diseases “Prof. Dr. Anton Mitov”, Faculty of Medicine, Medical University of Plovdiv, 4002 Plovdiv, Bulgaria; ivaylo.popov@mu-plovdiv.bg; 4 Clinic of Rheumatology, University Multi-Profile Hospital for Active Treatment “Kaspela”, Medical University of Plovdiv, 4001 Plovdiv, Bulgaria

**Keywords:** non-infectious uveitis, biological therapy, adalimumab, infliximab, tocilizumab, JAK inhibitors, uveitic macular oedema

## Abstract

Non-infectious uveitis (NIU) comprises a group of immune-mediated inflammatory diseases of the uvea that frequently affect people of working age. If inflammation is not controlled promptly, it may lead to permanent structural damage and visual loss. Prolonged corticosteroid use increases the risk of adverse effects. Contemporary treatment aims not only to control inflammation, but also to minimise dependence on corticosteroids. This narrative review examines the efficacy, safety, and place of biological and targeted therapies in the treatment of NIU. Adalimumab is the best-studied biological agent for intermediate, posterior, and panuveitis, as well as for uveitis associated with juvenile idiopathic arthritis. Infliximab has an important role in severe Behçet-associated uveitis and occlusive retinal vasculitis. Among the biological therapies with mechanisms of action other than TNF-α inhibition, tocilizumab is the best studied, particularly in refractory uveitic macular oedema. Janus kinase inhibitors (JAK inhibitors) are a rapidly developing class of targeted synthetic disease-modifying antirheumatic drugs (DMARDs). Clinical studies in NIU have shown variable efficacy and indicate their potential as a future therapeutic option. Treatment selection should be individualised and should take into account the anatomical form and severity of uveitis, the presence of systemic disease, macular oedema or occlusive retinal vasculitis, age and comorbidities, and the response to previous therapy. Therapeutic drug monitoring, biomarkers, and quantitative imaging parameters represent promising approaches to treatment personalisation; however, their clinical utility requires validation in prospective studies, and they are not currently part of routine clinical practice in non-infectious uveitis.

## 1. Introduction

Uveitis is an important preventable cause of permanent visual impairment, particularly among working-age individuals. The term “non-infectious uveitis” encompasses a heterogeneous group of immune-mediated inflammatory diseases, anatomically classified as anterior, intermediate, or posterior uveitis, or panuveitis; retinal vasculitis is often part of the clinical phenotype. The disease may be idiopathic or associated with a systemic immune-mediated condition, most commonly juvenile idiopathic arthritis (JIA), Behçet’s disease, spondyloarthritis, or sarcoidosis. Regardless of the aetiology, the primary clinical goal is to achieve rapid control of inflammation and maintain it for a sufficient period to reduce the risk of complications such as macular oedema, retinal ischemia, secondary glaucoma, and cataract.

Corticosteroids remain the first-line treatment for the initial control of inflammation. However, they are not an acceptable long-term solution in chronic or relapsing disease because of their numerous local and systemic adverse effects. The therapeutic goal is not merely temporary improvement but complete or satisfactory control with the lowest possible corticosteroid exposure. International recommendations from the Fundamentals Of Care for UveitiS (FOCUS) Initiative support the early initiation of systemic non-corticosteroid immunomodulatory therapy in persistent or relapsing inflammation, sight-threatening disease, or a need for prolonged treatment with high-dose corticosteroids [1,2]. Conventional corticosteroid-sparing agents—methotrexate, mycophenolate mofetil, azathioprine, ciclosporin, and tacrolimus—remain important first-line options. In some patients, however, they act too slowly, fail to provide adequate control, or cause substantial toxicity [1,3].

This is where biological and targeted therapies have a role. The two concepts are not entirely synonymous and should be distinguished. Biological disease-modifying antirheumatic drugs (bDMARDs) are large molecules produced through biotechnological processes. Most commonly, they are monoclonal antibodies or receptor proteins that block a specific cytokine, receptor, or cell population. Targeted therapy is a broader concept. In addition to biological agents, it includes targeted synthetic DMARDs (tsDMARDs)—small chemically synthesised molecules that enter cells and inhibit signalling enzymes, most notably Janus kinases (JAKs). This distinction has practical implications. Biological agents are administered subcutaneously or intravenously and may induce anti-drug antibodies. Small molecules are generally administered orally and affect several cytokine pathways simultaneously, but are associated with specific risks of systemic adverse effects [3,4,5].

## 2. Aim

The aim of this review is to present and analyse current evidence on the use of biological and targeted therapies in non-infectious uveitis (NIU), with emphasis on their mechanisms of action, clinical efficacy, safety, and place in the contemporary treatment algorithm, and to outline prospects for the development of individualised therapeutic strategies.

## 3. Methods

The present study is a structured narrative review rather than a systematic review. The literature search was conducted in PubMed/MEDLINE, Scopus, Web of Science, the Cochrane Library, and ClinicalTrials.gov and covered the period from January 2004 to August 2026. Earlier seminal publications were included only when necessary to provide relevant background on pathogenetic mechanisms. The search was last updated in August 2026.

The search strategy used combinations of the terms *noninfectious uveitis, intermediate uveitis, posterior uveitis, panuveitis, retinal vasculitis, uveitic macular oedema, juvenile idiopathic arthritis uveitis,* and *Behçet uveitis* with *biologic, targeted therapy, TNF, adalimumab, infliximab, golimumab, certolizumab, tocilizumab, sarilumab, rituximab, anakinra, canakinumab, gevokizumab, secukinumab, ustekinumab, abatacept, JAK inhibitor, tofacitinib, baricitinib, upadacitinib, filgotinib, brepocitinib,* and *therapeutic drug monitoring*. The search was supplemented by reviewing the reference lists of key publications and registries of ongoing clinical trials.

Publications were selected in two consecutive stages: (1) review of titles and abstracts and (2) full-text assessment when available. The initial search identified a total of 962 records. Given the narrative nature of the review, a formal PRISMA-based systematic screening of all identified records was not performed. The final synthesis included 72 priority-selected sources, comprising 50 full-text publications and 22 sources assessed on the basis of the available abstracts.

English-language publications involving patients with non-infectious uveitis were included if they provided data on the efficacy, corticosteroid-sparing effect, safety, or monitoring of biologic and targeted synthetic therapies, as well as their place in the therapeutic algorithm. Publications on infectious uveitis, exclusively preclinical studies without direct clinical applicability, duplicate analyses of the same study population, and reports providing insufficient information to assess therapeutic effects were excluded. Case reports and small case series were included only for emerging or uncommon therapeutic approaches when higher-level evidence was unavailable. Priority was given to clinical guidelines and recommendations, randomised controlled trials, systematic reviews, and meta-analyses, followed by prospective and retrospective real-world studies.

The strength of the evidence was assessed descriptively, without using the GRADE methodology. Evidence was considered “high” when consistent findings from more than one randomised trial were supported by long-term data; “moderate” when based on one randomised or prospective study with certain limitations and/or consistent observational data; “low” when derived primarily from retrospective cohort studies and case series; and “very low/preliminary” when based on small case series, individual case reports, early-phase studies, or incompletely published results. This assessment reflects the reliability of the available evidence rather than the direction of the observed effect. Therefore, a well-conducted randomised trial with a negative result may provide reliable evidence of a lack of clinical benefit.

## 4. Immunopathogenic Basis of Targeted Therapy

The eye has so-called immune privilege—a set of mechanisms that normally limit and suppress inflammation in order to protect delicate visual structures with limited regenerative capacity. This protection may nevertheless be disrupted. In such circumstances, the blood–retinal barrier becomes permeable to activated immune cells, which enter the eye and trigger inflammation. Once established, the inflammatory process may persist as a result of sustained immune activation. Experimental animal models and human data indicate that Th1 and Th17 lymphocytes, macrophages, and cytokines such as tumour necrosis factor alpha (TNF-α), IL-6, IL-1, IL-17, and IL-23 play an important role in the immune responses of patients with uveitis [4,5]. These molecules are therapeutic targets because they participate in different stages of the inflammatory process.

Tumour necrosis factor alpha is one of the principal pro-inflammatory cytokines; it facilitates leukocyte passage through the vascular wall and contributes to the maintenance of granulomatous and vasculitic inflammation. This role explains the rapid therapeutic effect of monoclonal anti-TNF antibodies in some severe forms of ocular inflammation, particularly Behçet-associated uveitis and retinal vasculitis. TNF-α inhibitors do not all provide the same degree of inflammatory control. Etanercept is a soluble TNF receptor protein and appears to be less effective for controlling ocular disease than monoclonal anti-TNF antibodies. In addition, cases of paradoxical onset or exacerbation of uveitis during treatment with etanercept have been described [6,7].

Another important mediator of inflammation is IL-6, which participates in the activation and regulation of various immune-cell populations. Increased IL-6 levels have been detected both in serum and in ocular fluids in different forms of NIU. IL-6 increases vascular permeability and contributes to the development of macular oedema. This explains the therapeutic interest in blocking the IL-6 signalling pathway, particularly in patients with uveitic macular oedema [6,7].

IL-1 is another important mediator of innate immunity. Its significance is best established in autoinflammatory syndromes and in some patients with Behçet’s disease. Nevertheless, IL-1 blockade has limited applicability and is not suitable for all forms of NIU [8].

The IL-23/Th17/IL-17 pathway plays an important role in the development of immune-mediated inflammation and has been extensively studied in diseases such as psoriasis and spondyloarthritis. This provides a rationale for investigating IL-17 blockade in non-infectious uveitis. Secukinumab is a monoclonal antibody that selectively blocks IL-17A and is established in the treatment of psoriasis and certain forms of spondyloarthritis. Despite its compelling mechanism of action, clinical studies in NIU have not demonstrated sufficient efficacy for controlling ocular inflammation or preventing relapses [9]. Consequently, secukinumab has not become standard therapy for NIU. These findings show that the successful modulation of a particular inflammatory mechanism in systemic disease does not necessarily produce the same therapeutic effect in ocular inflammation.

B-cells present antigens and produce autoantibodies and inflammatory mediators. Their suppression is therefore a rational approach in ANCA-associated vasculitis, IgG4-related diseases, and some refractory forms of uveitis. However, large randomised trials are lacking in the overall NIU population [10,11].

Janus kinase/signal transducer and activator of transcription (JAK/STAT) is an important intracellular signalling pathway through which several cytokines are involved in the inflammation act. JAK inhibitors block this pathway and may therefore simultaneously suppress the activity of several inflammatory mediators, including IL-6, interferons and IL-12/23. This may be advantageous in complex inflammatory diseases such as non-infectious uveitis. Their broader mechanism of action, however, is also associated with a risk of adverse effects, including infections, thrombosis, and cardiovascular events in predisposed patients [12,13].

## 5. Anti-TNF-α Therapy

### 5.1. Adalimumab—A Leading Agent in NIU

Adalimumab is a fully human monoclonal antibody against TNF-α. It is the only biological agent approved in the United States and the European Union for non-infectious intermediate and posterior uveitis and panuveitis in the relevant age groups. Its central role is supported not only by its mechanism of action, but also by a sequence of randomised trials, long-term follow-up, and persuasive data, including in children.

The international, randomised, double-masked, placebo-controlled phase III VISUAL I trial enrolled 217 adults with active non-infectious intermediate uveitis, posterior uveitis, or panuveitis. Patients received adalimumab or a placebo, while corticosteroids were gradually tapered according to a predefined regimen. In the adalimumab group, the time to treatment failure was significantly longer—24 weeks versus 13 weeks in the placebo group—corresponding to an approximately 50% reduction in the risk of treatment failure [14]. VISUAL II assessed the efficacy of adalimumab in patients with inactive but corticosteroid-dependent non-infectious uveitis. During corticosteroid withdrawal, adalimumab reduced the risk of uveitis flare or deterioration in visual acuity by 43% [15]. Long-term follow-up in VISUAL III showed that with continued adalimumab treatment, a substantial proportion of patients achieved control of ocular inflammation and reduced their need for corticosteroids. At week 150, 85% of patients remaining in the study had inactive uveitis. This result should nevertheless be interpreted cautiously because of the open-label design and attrition during follow-up [16,17,18].

The ADVISE study also provides important evidence by comparing adalimumab with conventional immunosuppressive therapy. Among the patients treated with adalimumab, inflammatory control and corticosteroid reduction were achieved more rapidly. By month 12, systemic corticosteroids had been successfully discontinued in 55% of patients receiving adalimumab compared with 40% of those receiving conventional immunosuppression [19]. These results do not mean that adalimumab should replace methotrexate or mycophenolate in all patients. They do, however, support earlier use in severe or sight-threatening inflammation, when rapid corticosteroid reduction is required, in bilateral posterior involvement, or when a systemic disease also requires anti-TNF therapy.

In children, the results are even more compelling. In the SYCAMORE study, which included patients with active juvenile idiopathic arthritis-associated uveitis despite methotrexate treatment, treatment failure occurred in 27% of patients receiving adalimumab compared with 60% in the placebo group [20]. The ADJUVITE study likewise confirmed the efficacy of adalimumab in chronic anterior uveitis inadequately controlled with topical corticosteroids and methotrexate [21]. These findings have been incorporated into recommendations for the treatment and monitoring of JIA-associated uveitis [22]. The ADJUST study addressed the important question of when adalimumab treatment can be discontinued. Within 48 weeks, treatment failure occurred in 68% of children after adalimumab discontinuation compared with only 14% of those who continued therapy [23]. These data indicate that the absence of clinical inflammation does not always represent sustained remission. Therefore, the decision to discontinue adalimumab should be individualised and followed by careful ophthalmological assessment.

When adalimumab is insufficiently effective, the cause should first be established. In primary non-response, inflammation remains active despite adequate treatment, necessitating a switch to an agent with a different mechanism of action. In secondary loss of response, the initial response is good, but the disease subsequently becomes active again. Possible causes include an inadequate dose, a low serum drug concentration, or the development of anti-adalimumab antibodies. In the event of unexplained exacerbation, therapeutic drug monitoring (TDM)—measurement of the drug concentration in the blood—may help determine whether the patient is receiving adequate drug exposure and why treatment may be ineffective. A low serum adalimumab level in the absence of antibodies may warrant shortening the dosing interval, whereas high antibody levels suggest switching agents. If disease remains active despite an adequate serum adalimumab level, treatment with an agent having a different mechanism of action should be considered [24,25]. There are still no universally accepted target serum adalimumab levels in uveitis; therefore, routine TDM for all patients is not recommended.

### 5.2. Infliximab and Other Anti-TNF Agents

Infliximab is a chimeric monoclonal anti-TNF antibody administered intravenously. The dose and interval between infusions can be adapted according to the clinical response, and the effect often develops rapidly. This makes infliximab a treatment of choice for severe Behçet-associated uveitis, particularly occlusive retinal vasculitis and other sight-threatening forms of posterior ocular inflammation. In 2026, EULAR published recommendations for the treatment of Behçet’s disease with monoclonal anti-TNF antibodies and recommended infliximab for severe posterior ocular involvement [26]. The main limitations are the need for intravenous infusions, the possibility of anti-drug antibody formation, and the risk of infusion reactions.

Comparative studies indicate a broadly similar efficacy of infliximab and adalimumab, despite differences in the diseases studied and in the treatment regimens used [27,28,29]. In Behçet’s disease, some studies report more rapid inflammatory control or a lower relapse risk with infliximab, whereas adalimumab offers more convenient subcutaneous administration and is suitable for long-term treatment [30]. The choice between the two agents should therefore be individualised. Infliximab is preferred in severe occlusive retinal vasculitis and when rapid control of inflammation is required, whereas adalimumab has a larger body of clinical evidence across different forms of NIU.

Golimumab and certolizumab pegol may be used as alternatives when other anti-TNF agents are ineffective or not tolerated, but evidence for their use in uveitis remains limited. Certolizumab pegol has minimal active placental transfer because it is a pegylated Fab′ fragment of a humanised monoclonal antibody against TNF-α and lacks an Fc fragment. This gives it an advantage when anti-TNF treatment is required during pregnancy, although evidence of its efficacy in active NIU is very limited.

Another agent is etanercept, which is not a monoclonal antibody but a recombinant soluble TNF-receptor fusion protein. It binds TNF and thereby limits its pro-inflammatory activity. It is not preferred for the treatment of uveitis because it provides less effective control of ocular inflammation than monoclonal anti-TNF antibodies. Cases of new-onset or exacerbation of uveitis during treatment have also been reported.

## 6. Alternative Biological and Targeted Therapies

### 6.1. IL-6 Receptor Blockade

Tocilizumab is a biological agent with demonstrated efficacy in ocular inflammation and a mechanism of action distinct from that of anti-TNF agents. The STOP-UVEITIS study enrolled 37 adults with active intermediate uveitis, posterior uveitis, or panuveitis who were treated intravenously with tocilizumab at 4 or 8 mg/kg every 4 weeks. At month 6, the investigators reported clinically meaningful improvement in 43.5% of patients in whom a reduction in vitreous haze could be assessed. Improvements in visual acuity, reduction in macular oedema, and limitation of angiographically visible vascular leakage were also observed [6].

It is well-established that IL-6 increases vascular permeability. This explains the efficacy of tocilizumab in uveitic macular oedema, including after failure of anti-TNF therapy [31]. In a multicentre study by Leclercq et al. involving 204 patients with NIU, inflammatory control at month 6 was achieved more frequently among patients treated with tocilizumab than among those receiving anti-TNF therapy. However, no significant differences between treatments were found in relapse frequency or change in visual acuity [7]. A 2025 review by Cao et al. also reported a favourable effect of tocilizumab, with the potential to reduce corticosteroid treatment and central retinal thickness [32]. The APTITUDE study investigated tocilizumab in patients with JIA-associated uveitis and an inadequate response to previous anti-TNF therapy. Tocilizumab produced a clinically meaningful therapeutic effect, although the prespecified threshold for treatment success was not reached [33]. These data establish tocilizumab as an important therapeutic option, particularly in persistent uveitic macular oedema, birdshot chorioretinopathy, JIA-associated uveitis after anti-TNF failure, and retinal vasculitis, although the agent is not approved for these indications.

RUBI is the first randomised study to directly compare adalimumab, anakinra, and tocilizumab in active refractory non-anterior NIU. At week 16, the prespecified efficacy criteria were met by 16% of patients receiving adalimumab and 14% of those receiving tocilizumab. The absence of macular oedema and retinal vasculitis was similar in the two groups, while the prednisone dose was reduced to ≤0.1 mg/kg/day in 59% and 74% of cases, respectively [8]. The low efficacy rates probably reflect both the strict inclusion criteria and the fact that participants had already proved difficult to treat. The study demonstrated similar efficacy of the two agents but did not establish the superiority of either.

The activity of sarilumab, another antibody directed against the IL-6 receptor, was investigated in the phase II SATURN study. Patient responses 16 weeks after treatment were compared with those in a placebo group. Evidence of efficacy was not conclusive, with improvement reported in 46.1% of patients compared with 30.0% in the placebo group [34]. Sarilumab should therefore be regarded as an alternative therapy that is not approved for NIU and cannot be considered equivalent to tocilizumab.

### 6.2. B-Cell-Directed Therapy

B-lymphocytes participate in the pathogenesis of numerous autoimmune and immune-mediated diseases, making B-cell-directed therapy a possible approach for severe and refractory forms of non-infectious uveitis. Rituximab is an anti-CD20 monoclonal antibody that reduces the number of B-cells without directly destroying them and may be a therapeutic option in selected severe and refractory forms of NIU, particularly in the presence of a B-cell-mediated systemic autoimmune disease. Published evidence consists mainly of retrospective series and individual case reports involving VKH, JIA-associated uveitis, Behçet’s disease, sarcoidosis, and retinal and systemic vasculitides. Investigators report a high rate of favourable responses, but the results are probably influenced by the preferential publication of successful cases and by the mixing of different diagnoses and treatment regimens. Larger prospective studies are therefore needed to define the efficacy and place of rituximab in NIU more accurately [10,11]. Treatment requires the assessment of hepatitis B status, immunoglobulin levels, infectious risk, and the anticipated need for repeated courses.

### 6.3. IL-1 Blockade

IL-1 blockade is a therapeutic approach aimed at limiting one of the key mediators of innate immune and autoinflammatory responses. Anakinra is a recombinant IL-1 receptor antagonist that blocks the activity of IL-1α and IL-1β, whereas canakinumab is a monoclonal antibody selectively directed against IL-1β. These agents may be appropriate in autoinflammatory diseases, including CAPS and Blau syndrome, and in selected cases of Behçet’s disease. Published studies show rapid suppression of ocular inflammation in some patients, but the response is not consistent. In the RUBI study, some patients received anakinra, but treatment was discontinued prematurely because of insufficient efficacy [8]. These results do not exclude potential benefit in selected patients in whom IL-1-mediated inflammation predominates.

Another member of this therapeutic group is gevokizumab, an allosteric monoclonal antibody against IL-1β. Initial observations in Behçet’s disease suggested promising results, but in the randomised EYEGUARD-B study, treatment did not significantly reduce the risk of ocular flare [35]. Accordingly, IL-1 blockade currently has a limited role in NIU and may be considered primarily in individual cases and carefully selected patients in whom IL-1 has a dominant pathogenic role.

### 6.4. IL-17/IL-23, T-Cell Co-Stimulation, and Other Approaches

In patients with spondyloarthritis and uveitis, the effect of biological therapy on systemic and ocular disease should be assessed separately when selecting treatment. For example, secukinumab, an IL-17 inhibitor, may control spondyloarthritis effectively. This does not mean, however, that it will control active uveitis. Three randomised studies of secukinumab (SHIELD, INSURE and ENDURE) did not demonstrate a statistically significant reduction in NIU relapses [9].

Biological agents targeting the IL-12/IL-23 signalling pathway are also of interest. Ustekinumab is a monoclonal antibody against the shared p40 subunit of IL-12 and IL-23, whereas guselkumab selectively blocks the p19 subunit of IL-23. Individual cases of a favourable effect on ocular inflammation have been described, particularly in patients with concomitant psoriasis or inflammatory bowel disease. These observations suggest a potential role for IL-23-directed therapy in selected forms of NIU, but the available evidence remains too limited to define its place in standard treatment.

Abatacept has a different mechanism of action; it is a fusion protein that suppresses T-lymphocyte activation by blocking the co-stimulatory signal required for activation. It has mainly been used in refractory JIA-associated uveitis and in birdshot chorioretinopathy. Published results are conflicting, and evidence of efficacy in NIU remains considerably more limited than that for adalimumab and tocilizumab.

## 7. Small Molecules and Novel Targeted Therapies

### 7.1. JAK Inhibitors

JAK inhibitors are oral targeted synthetic DMARDs rather than biological drugs, and they do not induce anti-drug antibodies. They simultaneously affect several cytokine pathways by inhibiting JAK1/2/3 or tyrosine kinase 2 (TYK2). The ability to modulate several inflammatory mechanisms at once makes JAK inhibition a potentially useful approach in NIU [12,13].

Among the JAK inhibitors, the most persuasive clinical evidence in NIU to date concerns filgotinib, an oral targeted agent with predominant activity against JAK1. Its efficacy was assessed in the randomised, placebo-controlled HUMBOLDT trial, which included 72 patients with active non-anterior NIU. At week 24, treatment failure occurred in 37.5% of patients receiving filgotinib 200 mg daily, compared with 67.6% in the placebo group [12]. Despite these favourable results, the study included few participants and was terminated early; the findings should therefore be interpreted cautiously.

Evidence is gradually accumulating for tofacitinib and upadacitinib. Tofacitinib predominantly inhibits JAK1 and JAK3, whereas upadacitinib is more selectively directed against JAK1. These agents have mainly been studied in patients with NIU in whom several previous therapies had failed to achieve adequate control [36]. Beckman et al. followed eight patients treated with JAK inhibitors; most achieved control of inflammation or a substantial reduction in flare frequency [37]. These findings are encouraging, but the small number of observations, absence of a control group, and concomitant use of other medicines preclude firm conclusions regarding efficacy.

Baricitinib is an oral JAK inhibitor with predominant activity against JAK1 and JAK2. Its efficacy in childhood uveitis was assessed in the JUVE-BRIGHT study, which included 29 children with active JIA-associated or chronic ANA-positive anterior uveitis; most had previously failed to achieve adequate control with anti-TNF therapy. At week 24, a therapeutic response was observed in 8 out of 24 patients treated with baricitinib (33.3%) [38]. These results indicate that JAK inhibitors do not have uniform efficacy across the different forms of NIU.

More encouraging preliminary results have been obtained with brepocitinib, an oral targeted agent that predominantly inhibits TYK2 and JAK1. In the phase II NEPTUNE study, a dose–response relationship was observed; at a dose of 45 mg, treatment failure by week 24 was reported in 29% of patients [39]. The efficacy of brepocitinib was also assessed in the phase III CLARITY study in active non-anterior NIU [40]. The evidence remains insufficiently persuasive, however, and brepocitinib should be regarded as an experimental rather than an established treatment for NIU.

Because evidence for the use of JAK inhibitors in ocular disease remains limited, they cannot currently be considered standard therapy for NIU. Nevertheless, the findings to date indicate their potential as a future therapeutic option, particularly in carefully selected patients with uncontrolled inflammation.

Patient safety is an important factor in treatment selection; therefore, the potential benefits of JAK inhibitors should be considered with particular caution. Adverse effects associated with this class include an increased risk of serious infections, such as reactivation of herpes zoster, venous thromboembolism, major adverse cardiovascular events, malignancy, and increased mortality in certain high-risk groups [41]. Risk is not uniform across all patients or all agents, so the decision to use these drugs must be individualised. Particular caution is warranted in patients aged ≥65 years, smokers, and those with cardiovascular disease, previous thrombotic events, or a history of malignancy. In such cases, the benefits and potential risks should be carefully weighed against available therapeutic alternatives.

### 7.2. Local Targeted Approaches

Local targeted therapy permits a high concentration of the drug in the eye with limited systemic exposure. Vamikibart is a monoclonal antibody directed at the IL-6 signalling pathway and is being developed for intravitreal treatment of uveitic macular oedema. Preliminary phase III results from the MEERKAT and SANDCAT studies indicate improvements in both visual acuity and central retinal thickness [42].

Another novel therapeutic approach is dazdotuftide (TRS01), a locally administered multitarget immunomodulator being developed for the treatment of non-infectious anterior uveitis. Phase III data indicate an anti-inflammatory effect. The results are not sufficiently persuasive, however, to support a definitive conclusion regarding efficacy [43].

Local targeted therapies cannot replace systemic treatment in patients with bilateral uveitis and active systemic disease. Nevertheless, they are valuable options in ocularly limited forms of NIU, uveitic macular oedema, or in patients for whom systemic immunosuppression is contraindicated or associated with increased risk.

## 8. Treatment Selection in Clinical Practice

Selection of a specific treatment requires the prior assessment of several important factors: how rapidly the inflammation must be controlled; which ocular structures and visual functions are immediately threatened; whether a concomitant systemic disease also requires treatment; and the reason for failure of previous therapy.

### 8.1. When to Escalate to Biological or Targeted Therapy

Biological or targeted therapy is most often required when inflammation is inadequately controlled with conventional treatment or when factors associated with a high risk of visual loss are present: persistent activity despite adequate conventional immunosuppression; inability to discontinue prednisone or reduce it to ≤7.5 mg/day without a new flare; recurrent sight-threatening inflammation; occlusive retinal vasculitis; progressive visual loss or structural damage; bilateral posterior-segment involvement; intolerance or contraindication to conventional agents; severe JIA-associated uveitis after methotrexate treatment; or a systemic disease that can be treated with the same biological agent [1,3]. In these situations, biological therapy should not be delayed until all conventional DMARDs have been sequentially exhausted. Conversely, in mild, unilateral and well-controlled anterior uveitis, the systemic risk of biological therapy is not justified. Treatment should therefore be individualised according to the severity and location of inflammation, the risk to vision, and the presence of systemic disease. In this context, local agents, conventional DMARDs and targeted systemic therapy are not mutually exclusive but complementary therapeutic options.

### 8.2. Therapeutic Target and Assessment of Response

The principal therapeutic goal is control of inflammation rather than partial clinical improvement alone. This entails no new chorioretinal or vascular lesions, minimal or absent cells in the anterior chamber and vitreous haze, no active angiographic leakage, and resolution or substantial reduction in uveitic macular oedema. Successful treatment should also be accompanied by stabilisation or improvement of visual acuity and discontinuation or minimisation of systemic corticosteroids.

Assessment of therapeutic effect should not rely solely on visual acuity but should combine clinical examination with imaging, particularly optical coherence tomography (OCT) and fluorescein angiography in retinal vasculitis. Visual acuity may remain good despite active peripheral vasculitis, or conversely, may remain poor after inflammation has been controlled because of irreversible structural changes.

The first comprehensive assessment of treatment response is usually performed between the second and fourth month after treatment initiation, depending on disease severity and the drug used. In the event of a partial or inadequate response, possible causes should be sought, including poor adherence, an inadequate dose or inappropriate dosing interval, unrecognised infection or masquerade syndrome, a structural cause of reduced vision, or insufficient drug exposure. Treatment modification should be considered if a new sight-threatening lesion develops, retinal vasculitis or uveitic macular oedema persists, corticosteroids cannot be tapered, unacceptable toxicity occurs, or secondary loss of therapeutic effect is demonstrated.

### 8.3. Practical Treatment Algorithm

Contemporary NIU treatment involves several sequential steps aimed at rapidly controlling inflammation, limiting corticosteroid exposure, and promptly switching to more effective therapy when the response is inadequate.

Initial control of inflammation is achieved with local or systemic corticosteroids, depending on the anatomical location and severity of uveitis. A subsequent treatment strategy that will allow gradual tapering, and where possible, discontinuation, should be planned from the outset. Methotrexate, mycophenolate, or azathioprine may be used as first-line corticosteroid-sparing therapy; the choice depends on the characteristics of ocular inflammation, the concomitant systemic disease, and the individual patient profile.

Biological therapy may be initiated before the options for conventional immunosuppressive treatment have been exhausted, particularly when there is a high risk of irreversible visual impairment. Adalimumab has an established role in active or corticosteroid-dependent non-anterior NIU and in JIA-associated uveitis, whereas infliximab is particularly suitable for severe ocular involvement in Behçet’s disease, including occlusive retinal vasculitis.

In the event of an inadequate response to anti-TNF therapy, it should first be determined whether the agent has been administered at an adequate dose and whether sufficient drug exposure has been achieved; only then should a switch to another anti-TNF agent or to treatment with a different mechanism of action be considered.

The choice of subsequent therapy is largely determined by the dominant clinical finding and the concomitant systemic disease. Tocilizumab is preferred in refractory uveitic macular oedema, whereas rituximab may be considered in selected B-cell-mediated or systemic vasculitic diseases. JAK inhibitors represent a newer therapeutic option in repeatedly refractory disease, but because the available evidence remains limited, their use in NIU requires particular caution (Table 1).

### 8.4. Potential Dosing Regimens, Routes of Administration, and Treatment Duration

For biological and targeted therapies for non-infectious uveitis (NIU), dosing regimens should be regarded as indicative, since many of these agents are used off-label, while regimens for newer molecules are based on clinical trial protocols. Treatment duration is determined not solely by the number of administrations, but also by the achievement of objective inflammatory control, the ability to reduce corticosteroid therapy, and the safety profile. An early assessment is usually performed within 8–16 weeks, whereas for slower-acting or intermittently administered agents, assessment may be extended to 3–6 months. Potential regimens are summarised in Table 2.

## 9. Safety, Screening and Monitoring

Safety is an important component of treatment selection. Before therapy is initiated, it should be confirmed that the uveitis is non-infectious and that the patient has no active systemic infection. The minimum assessment includes a medical history and tuberculosis testing, chest imaging when indicated, screening for hepatitis B (HBsAg, anti-HBc, anti-HBs) and hepatitis C (HCV), human immunodeficiency virus (HIV) serology when clinically indicated, a full blood count, liver function tests, creatinine, vaccination status, possible pregnancy, and a history of malignancy and cardiovascular disease [44,45]. Necessary immunisation with inactivated vaccines should preferably be completed before immunosuppression is started, whereas live vaccines are generally avoided during substantial biological or targeted therapy.

With anti-TNF therapy, the principal risks are serious infections and reactivation of latent tuberculosis or hepatitis B infection, as well as local or infusion reactions. Less commonly, paradoxical immune reactions, exacerbation of demyelinating disease, and worsening of severe heart failure may occur. Immunogenicity is more pronounced with infliximab, and anti-drug antibodies may lead to reduced or lost therapeutic effect; concomitant methotrexate or another immunomodulator may therefore be appropriate, although this increases systemic immunosuppression. Adalimumab has a similar safety profile, characteristic of anti-TNF therapy [47].

During tocilizumab treatment, neutrophil and platelet counts, liver function tests (ALT/AST) and the lipid profile should be monitored periodically. Particular attention should be paid to the risk of serious infection and gastrointestinal perforation, especially in patients with diverticulitis. Tocilizumab may suppress the rise in C-reactive protein, so laboratory parameters do not always reflect the presence or severity of infection. Clinical symptoms and the patient’s general condition are therefore of primary importance during follow-up [48].

The principal adverse effects of rituximab are infusion reactions, hepatitis B reactivation, late-onset neutropenia, hypogammaglobulinaemia, and rare severe opportunistic infections; hepatitis B screening is mandatory [49].

For patients receiving JAK inhibitors, assessment of the risk of venous thromboembolism and cardiovascular events is required in addition to a full blood count, transaminases, and lipid measurements. Smoking, a history of malignancy, and the risk of herpes zoster infection are relevant considerations. In a high-risk patient for whom an equally effective, safer alternative is available, a JAK inhibitor is not recommended [41].

Monitoring should be both systemic and ophthalmological. At treatment initiation, the full blood count and liver function tests are usually checked 4–8 weeks later, and if stable, every 3–6 months. The frequency of eye examinations depends on disease activity. During a flare or corticosteroid taper, review is generally scheduled every 2–6 weeks. In stable remission, the interval is extended and individualised.

## 10. Discussion

The available literature indicates a clear hierarchy of evidence but does not support a universal therapeutic algorithm. Adalimumab has the strongest evidence base, supported by several randomised trials in adults and children, long-term follow-up, and real-world data. For infliximab, the evidence is moderate and is derived primarily from comparative and observational studies; however, its clinical efficacy has consistently been supported in severe Behçet’s uveitis and occlusive retinal vasculitis. Tocilizumab is also supported by a moderate level of evidence, particularly in refractory uveitic macular oedema and following failure of anti-TNF therapy. The evidence for rituximab, IL-1 blockade, IL-12/23–IL-23-targeted therapies, and abatacept is low or very low and does not support their routine use outside carefully selected clinical phenotypes. For JAK inhibitors, the evidence should be assessed on an agent-specific basis: filgotinib is supported by randomised trial data, although with methodological limitations, whereas evidence for the other agents remains predominantly preliminary or observational.

Results from newer studies are gradually changing some traditional concepts regarding treatment sequencing. ADVISE showed that in appropriately selected patients, early introduction of biological therapy may permit more rapid corticosteroid reduction [19]. This does not diminish the importance of conventional DMARDs, which remain an important part of treatment, but indicates that in patients at high risk of visual loss it may be inappropriate to wait until all conventional agents have been tried. RUBI, in turn, found a similar short-term effect of adalimumab and tocilizumab in highly treatment-refractory patients, whereas anakinra did not demonstrate sufficient efficacy [8]. The ADJUST data raise another important practical question: achieving disease control does not necessarily mean that treatment can be discontinued [23].

Interpretation of these findings must also take into account the limitations in the available evidence base. NIU comprises a rare and highly heterogeneous group of diseases, so clinical trials often include small numbers of patients with different diagnoses and characteristics. The criteria used to assess therapeutic effect also differ. Vitreous haze, for example, has limited sensitivity, while visual acuity may remain permanently reduced despite control of inflammation because irreversible structural changes have already occurred. Gradual corticosteroid tapering creates an additional challenge: although it is an important therapeutic goal, it may influence treatment success in some studies. Real-world data should also be interpreted cautiously, as newer therapies are generally used in patients with more severe and refractory disease. Because of these differences, response rates from separate studies should not be compared directly without considering the characteristics of the enrolled patients and the assessment criteria used.

In addition to efficacy and safety, treatment selection is influenced by cost, access to therapy and the availability of biosimilars. Biosimilar forms of adalimumab and infliximab may improve access to biological treatment and reduce its cost. If disease control is lost after a stable patient is switched to a biosimilar, the cause should be investigated carefully. Adherence, correct administration, drug levels, and anti-drug antibodies, as well as natural fluctuations in disease activity, should be assessed. Worsening after switching to a biosimilar should therefore not automatically be interpreted as evidence of lower efficacy of the product itself.

## 11. Future Perspectives

Current uveitis classification primarily describes the location of inflammation but does not explain why individual patients respond differently to anti-TNF, IL-6, or JAK blockade. Future approaches will probably combine the clinical phenotype and systemic disease with HLA characteristics, cytokine profiles, gene activity, and the quantitative imaging parameters. The aim is not to identify a single universal biomarker, but rather a combination of markers that can support selection of the most appropriate therapeutic mechanism.

Therapeutic drug monitoring in anti-TNF therapy is closest to routine implementation. Determination of serum drug concentrations and anti-drug antibodies may assist decisions about increasing drug exposure, switching to another agent in the same class, or selecting a medicine with a different mechanism of action [24,25]. Prospective studies are nevertheless needed to define optimal concentrations, the appropriate timing of testing, and the cost-effectiveness of this approach.

Future studies should use more precise and standardised assessment criteria, including quantitative OCT parameters and wide-field angiography. Automated analysis of retinal morphology and vascular leakage may assist in detecting subclinical activity and individualising treatment.

The optimal duration of biological therapy also remains uncertain. Gradual treatment de-escalation after a sufficiently prolonged corticosteroid-free remission and in the absence of clinical or imaging signs of activity is probably the more appropriate approach. In the future, biomarkers of “durable remission” may support decisions about when treatment can be safely reduced or discontinued.

## 12. Conclusions

Biological and targeted therapy in NIU should not be regarded simply as a last resort after all other options have been exhausted. It is part of an individualised therapeutic approach in which treatment selection is determined by the clinical characteristics of uveitis, the concomitant systemic disease, and the reasons for failure of previous therapy. Adalimumab remains the agent with the most persuasive clinical-trial support, whereas infliximab has an important role in selected forms of sight-threatening Behçet-associated uveitis. Tocilizumab is a particularly valuable option in refractory uveitic macular oedema. Rituximab, IL-1 blockade, and other biological agents may be appropriate in carefully selected patients, but evidence for their efficacy remains more limited. JAK inhibitors expand the options for targeted therapy beyond biological agents and have shown promising results, but they are not yet part of standard NIU treatment.

An effective therapeutic approach should be dynamic and tailored to the individual patient. It begins with an assessment of the risk to vision and the definition of a clear, measurable therapeutic goal, followed by the timely initiation of treatment that permits limitation of corticosteroid exposure. When the response is inadequate, the cause should be established before treatment is changed. Concomitant systemic diseases and the individual risk profile must also be considered. Future progress will probably depend less on the arrival of yet another medicine than on the ability to determine which patient will benefit most from which therapeutic approach, at what point, and for how long.

## Figures and Tables

**Table 1:** Mechanism of action and clinical use of biological and targeted agents [6,7,8,9,10,11,12,13,14,15,17,19,20,24,25,26,27,28,29,30,31,32,34,35,36,37,38,39,40,41,42,44,45,46].

Agent	Molecular Target	Mechanism	Clinical Indications
**Adalimumab**	TNF-α	Monoclonal antibody; neutralises free and membrane-bound TNF-α	Active/inactive intermediate and posterior uveitis, panuveitis; JIA-associated uveitis; Behçet-associated uveitis
**Infliximab**	TNF-α	Chimeric monoclonal antibody; intravenous administration and rapid effect	Sight-threatening Behçet-associated uveitis, occlusive retinal vasculitis, severe refractory NIU
**Golimumab, certolizumab**	TNF-α	Monoclonal antibody or antibody-fragment TNF inhibitors	Intolerance or failure of other anti-TNF agents; systemic indication
**Etanercept**	TNF receptor	Soluble TNF-receptor fusion protein	Not preferred for active ocular inflammation
**Tocilizumab**	IL-6R	Blocks the soluble and membrane-bound IL-6 receptor	Refractory UME, anti-TNF failure, birdshot chorioretinopathy, JIA-associated uveitis
**Sarilumab**	IL-6R	Blocks the IL-6 receptor	Refractory posterior NIU
**Rituximab**	CD20	Reduces CD20-positive B cells	ANCA- or IgG4-related disease, VKH, refractory NIU
**Anakinra, canakinumab**	IL-1R/IL-1β	IL-1 receptor or IL-1β blockade	Autoinflammatory syndromes; selected Behçet phenotypes
**Gevokizumab**	IL-1β	Allosterically modulates IL-1β	Behçet-associated uveitis
**Secukinumab**	IL-17A	Neutralises IL-17A	Systemic SpA/PsA; uncertain efficacy in active NIU
**Ustekinumab and others**	IL-12/23 or IL-23	Blocks p40 or IL-23	Rare cases with IBD/psoriasis
**Abatacept**	CD80/86–CD28	Suppresses T-cell co-stimulation	Refractory JIA-associated uveitis, birdshot chorioretinopathy
**Filgotinib**	JAK1	Oral selective JAK1 inhibitor	Active non-anterior NIU
**Baricitinib, tofacitinib, upadacitinib**	JAK1/2, JAK1/3	Block multiple cytokine signals	Refractory NIU; JIA/SpA phenotype
**Brepocitinib**	TYK2/JAK1	Oral dual-kinase inhibition	Active non-anterior NIU
**Vamikibart**	Local IL-6	Intravitreal anti-IL-6 antibody	Uveitic macular oedema

Abbreviations: TNF—tumour necrosis factor; IL—interleukin; JAK—Janus kinase; CD—cluster of differentiation; TYK—tyrosine kinase; JIA—juvenile idiopathic arthritis; NIU—non-infectious uveitis; UME—uveitic macular oedema; ANCA—antineutrophil cytoplasmic antibodies; VKH—Vogt–Koyanagi–Harada disease; SpA—spondyloarthritis; PsA—psoriatic arthritis; IBD—inflammatory bowel disease.

**Table 2:** Potential dosing regimens, routes of administration, and approximate treatment duration of biological and targeted therapies [1,2,5,9,12,13,23,34,36,37,38,39,40,41,42,43,47,48,49].

Drug	Potential Dosing Regimen	Route of Administration	Treatment Duration and Assessment
**Adalimumab**	Adults: initial dose of 80 mg, followed by 40 mg after 1 week, then 40 mg every 2 weeks. In children, the dose depends on body weight; usually 20 mg every 2 weeks for 15–<30 kg and 40 mg every 2 weeks for ≥30 kg.	Subcutaneous	Assessment after 8–16 weeks. Dose tapering or treatment withdrawal may be considered after prolonged stable remission; in JIA-associated uveitis, often after ≥2 years of inactive disease.
**Infliximab**	Usually 5 mg/kg at weeks 0, 2, and 6, followed by administration every 4–8 weeks; in severe refractory ocular inflammation, doses up to 10 mg/kg may be used.	Intravenous	Early assessment. There is no fixed treatment duration; therapy is continued until sustained control is achieved and gradual extension of dosing intervals becomes possible.
**Golimumab**	50 mg once every 4 weeks; its use in NIU is off-label.	Subcutaneous	Assessment after 3–6 months. No specific treatment duration has been established for uveitis; treatment should be continued only when there is a convincing clinical benefit.
**Certolizumab pegol**	400 mg at weeks 0, 2, and 4, followed by 200 mg every 2 weeks or 400 mg every 4 weeks; the regimen is off-label for NIU.	Subcutaneous	Assessment after 3–6 months; there is no validated treatment duration for NIU. The decision should be based on the ocular and systemic response.
**Etanercept**	50 mg once weekly in adults; the paediatric dose is weight-based.	Subcutaneous	Not recommended for the treatment of active ocular inflammation because of lower efficacy and the risk of paradoxical uveitis.
**Tocilizumab**	4–8 mg/kg every 4 weeks (maximum 800 mg per infusion) or 162 mg subcutaneously once weekly; for certain systemic indications, the subcutaneous dosing interval may be every 2 weeks.	Intravenous or subcutaneous	Assessment at 3–6 months, including OCT in cases of macular oedema. There is no fixed treatment duration; therapy may be continued when effective and acceptably safe.
**Sarilumab**	200 mg every 2 weeks; in cases of laboratory abnormalities or intolerance, the dose is reduced to 150 mg every 2 weeks.	Subcutaneous	In NIU, it has been studied only in the short-term. No established maintenance duration is available, and it is not considered standard therapy.
**Rituximab**	1000 mg on days 1 and 15, with a repeat course after 6 months; alternatively, 375 mg/m^2^ once weekly for 4 consecutive weeks.	Intravenous	Repeat courses are determined by clinical activity, systemic disease, B-cell reconstitution, and immunoglobulin levels.
**Anakinra**	100 mg daily	Subcutaneous	Assessment after 8–12 weeks. Treatment is discontinued if there is no clear benefit; in responders, it may be administered continuously, with no established NIU-specific treatment duration.
**Canakinumab**	150 mg every 6–8 weeks	Subcutaneous	Assessment after 2–3 administrations. There is no standardised treatment duration for NIU.
**Gevokizumab**	30 or 60 mg every 4 weeks	Subcutaneous or intravenous	Only within clinical trials.
**Secukinumab**	For systemic indications: 150–300 mg at weeks 0, 1, 2, 3, and 4, followed by administration every 4 weeks.	Subcutaneous	There is no standard treatment duration for NIU because of insufficient ocular efficacy.
**Ustekinumab/Guselkumab**	Ustekinumab: 45 mg at weeks 0 and 4, followed by every 12 weeks; Guselkumab: 100 mg at weeks 0 and 4, followed by every 8 weeks.	Subcutaneous	No established regimen or treatment duration for NIU.
**Abatacept**	125 mg once weekly or 10 mg/kg intravenously at weeks 0, 2, and 4, followed by every 4 weeks.	Subcutaneous or intravenous	Assessment after 3–6 months. There is no fixed treatment duration for NIU.
**Filgotinib**	200 mg once daily.	Oral	Experimental therapy in NIU.
**Baricitinib**	2 mg daily in children younger than 6 years and 4 mg daily in older children.	Oral	Assessment at week 24.
**Tofacitinib**	5 mg twice daily or 11 mg extended-release once daily; paediatric regimens are weight-based.	Oral	Assessment after 8–16 weeks.
**Upadacitinib**	15 mg once daily.	Oral	Assessment after 8–16 weeks.
**Brepocitinib**	15 or 45 mg once daily in clinical trials.	Oral	Experimental treatment.
**Vamikibart**	0.25 or 1 mg intravitreally every 4 weeks for four initial doses, followed by administration as needed.	Intravitreal	Treatment duration is defined by clinical trial protocols; there is no approved routine regimen.
**Dazdotuftide (TRS01)**	1% ophthalmic solution, one drop four times daily.	Topical, ophthalmic drops	Studied as a short-term treatment for active anterior NIU.

Note: The regimens are indicative and are derived from approved systemic regimens or ophthalmic studies. With the exception of adalimumab for selected forms of NIU, most uses are off-label or experimental.

## Data Availability

No new data were created or analysed in this study. Data sharing is not applicable to this article.
